# Neuroimaging in psychedelic drug development: past, present, and future

**DOI:** 10.1038/s41380-023-02271-0

**Published:** 2023-09-27

**Authors:** Matthew B. Wall, Rebecca Harding, Rayyan Zafar, Eugenii A. Rabiner, David J. Nutt, David Erritzoe

**Affiliations:** 1 Invicro, London, UK; 2 Faculty of Medicine, Imperial College London, London, UK; 3 Centre for Psychedelic research and Neuropsychopharmacology, Imperial College London, London, UK; 4 Clinical Psychopharmacology Unit, Faculty of Brain Sciences, University College London, London, UK

**Keywords:** Neuroscience, Depression

## Abstract

Psychedelic therapy (PT) is an emerging paradigm with great transdiagnostic potential for treating psychiatric disorders, including depression, addiction, post-traumatic stress disorder, and potentially others. ‘Classic’ serotonergic psychedelics, such as psilocybin and lysergic acid diethylamide (LSD), which have a key locus of action at the 5-HT2A receptor, form the main focus of this movement, but substances including ketamine, 3,4-Methylenedioxymethamphetamine (MDMA) and ibogaine also hold promise. The modern phase of development of these treatment modalities in the early 21st century has occurred concurrently with the wider use of advanced human neuroscientific research methods; principally neuroimaging. This can potentially enable assessment of drug and therapy brain effects with greater precision and quantification than any previous novel development in psychiatric pharmacology. We outline the major trends in existing data and suggest the modern development of PT has benefitted greatly from the use of neuroimaging. Important gaps in existing knowledge are identified, namely: the relationship between acute drug effects and longer-term (clinically-relevant) effects, the precise characterisation of effects at the 5-HT2A receptor and relationships with functional/clinical effects, and the possible impact of these compounds on neuroplasticity. A road-map for future research is laid out, outlining clinical studies which will directly address these three questions, principally using combined Positron Emission Tomography (PET) and Magnetic Resonance Imaging (MRI) methods, plus other adjunct techniques. Multimodal (PET/MRI) studies using modern PET techniques such as the 5-HT2A-selective ligand [11 C]Cimbi-36 (and other ligands sensitive to neuroplasticity changes) alongside MRI measures of brain function would provide a ‘molecular-functional-clinical bridge’ in understanding. Such results would help to resolve some of these questions and provide a firmer foundation for the ongoing development of PT.

## Introduction

The use of psychoactive substances for medicinal or spiritual purposes stretches back into pre-history [1], with the first recorded study of psychedelics in a clinical context carried out on mescaline in the early 20th Century. However, the wide-spread clinical use of psychedelics began in the late 1940s [2] following the first synthesis of lysergic acid diethylamide (LSD) by Albert Hoffmann in 1943. This work occurred alongside the discovery of other key classes of psychiatric drugs such as the neuroleptics (e.g., Chlorpromazine, first synthesized in 1951 [3]) and Mono-Amine Oxidase Inhibitor (MAOI) anti-depressants (first recognized for their mood-elevating effects in 1952 [4], which collectively led to a new focus on biological mechanisms in psychiatry [5]. In the 1940s psychedelics were often termed ‘psychotomimetics’ and were thought to mimic the symptoms of psychiatric conditions, principally schizophrenia. Interest in their therapeutic potential grew throughout the 1950s, with large-scale use in the United States, the United Kingdom and the Czech Republic [2] for a number of psychiatric conditions. By 1961, more than 1000 scientific articles on LSD had been published [6]. However, growing concern about the recreational use of LSD, and its (perceived) links to the anti-Vietnam war protests and general counter-culture movement, led to it being banned in the US in the mid 1960s. Most countries worldwide followed suit, encouraged by President Nixon’s repressive “war on drugs”. Climactically, the 1971 United Nations Convention on Psychotropic Drugs and the Misuse of Drugs Act 1971 placed psychedelics into Schedule 1/Class A. This implied they did not have any known therapeutic potential and were considered to be highly addictive [7]. Concurrently, psychedelic treatment models clashed somewhat with the growing emphasis on randomized controlled trials as the gold standard for proving treatment efficacy [8]. These factors effectively halted clinical and mechanistic research into psychedelics for nearly 50 years [9].

The technology available for neuroscientific research in the 1950s was comparatively primitive by modern standards. While initial electroencephalography (EEG) studies using LSD were conducted in humans [10], there was relatively little opportunity (in terms of both time, and the technology available) to carry out any further neuroimaging work before the prohibition of the 1970s. This led to a number of stagnant decades in the development of our understanding of the neural mechanisms underpinning the effects of psychedelics in humans. Although the main pharmacological mode of action of classical psychedelics as 5-HT2 receptor agonists was posited in the mid-1980s [11], and confirmed with the use of the 5-HT2 antagonist ketanserin [12] and the development of 5-HT2A receptor (5-HT2AR) knockout mice [13], translational neuropsychopharmacological work in humans was strongly limited by regulatory difficulties until very recently [14].

The resurgence of clinical research into psychedelics in the early 21st Century has developed in a markedly different context with the increased availability of sophisticated research technologies. During the interlude in research efforts, impressive developments in molecular, structural, and functional imaging techniques of the human brain have occurred. Modern work has sought to apply these novel technologies to the study of the ‘classic’ psychedelics (LSD, psilocybin; a pro-drug that is metabolized to the active substance psilocin, and N,N-Dimethyltryptamine; DMT) and compounds with related or similar subjective effects (though different pharmacological profiles), such as 3,4-Methylenedioxy methamphetamine (MDMA), ketamine, and ibogaine.

## Acute challenge studies

While some early clinical trial work in the mid-2000s tested MDMA in post-traumatic stress disorder [15], most of the initial studies in the modern era focused on safety, tolerability, and evaluation of acute effects in healthy subjects by harnessing the power of modern neuroscience methods. These studies mostly used functional Magnetic Resonance Imaging (fMRI) methods, with some ancillary work using Magnetoencephalograhy (MEG) or Electroencephalography (EEG). Resting-state data from these studies has provided crucial insights into the acute brain-network effects of compounds like psilocybin [16, 17], LSD [18, 19], and MDMA [20]. A key discovery has been that classic psychedelics have a profound impact on the normal large-scale network patterns of brain connectivity, leading to a severe disruption of the network structure [19, 21, 22]. These findings have formed the foundation of modern theories of how psychedelics exert both their acute and longer-term effects, e.g., the relaxed-beliefs under psychedelics or REBUS model [23], and the cortico-striatal thalamo-cortical or CSTC model [24–26]. In addition, task fMRI data from these studies have provided vital insights with potential clinical relevance, such as the effect of LSD on the brain’s response to positive hedonic stimuli such as music [27, 28], effects of psilocybin on social and emotional processing [29] and the effects of MDMA on the recall of positive and negative emotional memories [30].

Molecular imaging of the direct action of psychedelics with Positron Emission Tomography (PET) has been much less exploited. This is likely due to the increased associated costs, greater ethical and methodological difficulties (e.g. invasive procedures, more medical supervision required, fewer research-grade PET facilities), and, most crucially, the lack of 5-HT2AR agonist PET radioligands most suitable for evaluation of the pharmacological effects of agonist drugs. The utility of radioligands such as [18F]setoperone, [18F]altanserine and [11C]MDL100907 was limited by the methodological issues inherent in the evaluation of agonist compounds using antagonist PET radioligands [31].

The 5-HT2AR agonist PET ligand [11C]Cimbi-36 is relatively new [32–34], has higher sensitivity to serotonin receptor agonists and has so far been used at only two PET imaging sites [35, 36]. [11C]Cimbi-36 has been used to demonstrate that the acute subjective effects of psilocybin (or rather its active metabolite psilocin) are related to its binding at 5-HT2A receptors [36]. Downstream effects of psilocybin were evaluated previously using PET ligands such as [11C]raclopride, to evaluate changes in dopamine release [37] and [18F]fluorodeoxyglucose PET [38] to evaluate changes in glycolysis and brain metabolism. A recent study examined the relationship between 5-HT2AR binding with PET and long-term changes in personality factors [39], and the same team also reported neocortical 5-HT2AR binding to be negatively associated with peak plateau duration of the psilocybin experience [40]. PET investigations have also helped understand long-term effects on serotonin brain markers, such as serotonin transporters and 5-HT2A receptors, following different degrees of recreational use of MDMA and psychedelics [41].

## Neuroimaging in clinical trials

Inspired both by the historical reports of positive clinical effects and results from modern acute challenge studies, researchers began to investigate the potential of psychedelics in patient groups, often with neuroimaging as an adjunct to the main clinical trial outcomes. Again, (f)MRI has been the usual method of choice in these studies because of its relatively low-cost and non-invasive nature, with neuroimaging measures usually used as an objective index of treatment effects, i.e., using scans conducted before and after treatment rather than during acute dosing. Initial studies in psilocybin for treatment-resistant depression [42] and major depression [43, 44] have used this approach, with other similar trials now underway evaluating the therapeutic potential of psychedelics in various psychiatric disorders including anorexia nervosa, Obsessive-Compulsive Disorder (OCD), Post-Traumatic Stress Disorder (PTSD), chronic pain, and addiction.

These studies have found that a number of changes (i.e. identified by comparing before vs. after treatment) in brain function can be meaningfully related to clinical outcomes. The first open-label study in treatment-resistant depression patients [42] showed that changes in cerebral blood flow (measured with arterial spin-labelling MRI) in the amygdala were correlated with changes in depression scores [45]. Differences in the functional connectivity of the medial pre-frontal cortex and hippocampus were also identified following treatment with psilocybin in the same patient group. Further work from the same cohort has found increased responses to emotional stimuli in the amygdala [46], that changes in amygdala connectivity are predictive of some clinical outcomes [47], and that patients show an increased brain response to music stimuli following treatment [48]. Analogous effects (changes in neural emotional processing, increased positive affect, reduced anxiety) have also been reported in a small group of healthy volunteers with one-week and one-month follow-up assessments [49].

More recent work has shown decreases in brain modularity (the tendency of the brain to function in well-defined networks). Modularity was assessed in two groups; patients from the initial open-label study [42] and patients from a double-blind study where psilocybin was compared with the selective serotonin reuptake inhibitor (SSRI) escitalopram [44]. Changes in modularity produced by psilocybin were related to change in clinical outcome scores in both studies, while escitalopram produced no such effects [50]. Despite some critiques of the statistical methods used [51] these findings are broadly consonant with the disruptive effects on network function seen in the acute-challenge studies (plus increased connectivity seen in healthy subjects over longer time-scales; [49], and suggest that these effects might be central to psilocybin’s rapid anti-depressant effects [52]. Another open-label study in patients with major depressive disorder reported increases in cognitive (measured by perseverative errors on a set-shifting task) and neural (as seen by the dynamics of functional connectivity via fMRI) flexibility which persisted up to four weeks post-treatment, as well as reductions in glutamate and N-Acetylaspartate concentrations (using Magnetic Resonance Spectroscopy; MRS) in the anterior cingulate cortex [53].

Results from these (albeit few and relatively small) trials are necessarily varied, as they have used different methods, analysis approaches, and endpoints. One recent review of resting-state psychedelic work [54] has made the points that there is a great variety of analysis methods in the literature, and over half the published corpus consists of analyses of just two datasets. More studies, larger, well-conducted clinical trials, standardisation of methods, and independent replication of the effects described so far are clearly badly needed. It remains to be seen if a reliable imaging biomarker of the clinical response can be identified, though the global effects on brain networks may be a potential candidate in biomarker research efforts [52, 55, 56]. Additionally, there is a current viewpoint that putative increases in neuroplasticity likely underlie positive clinical effects of psychedelic therapy [52, 57, 58], which is largely derived from pre-clinical work (for a recent review see [59]. Neuroplasticity is a broad term that can encompass several identified mechanisms from molecular processes to large-scale brain activity dynamics. So far, the only evidence of a possible neuroplastic effect of psychedelics in humans have been with low-dose LSD [60] and ayahuasca [61]. Both studies which showed increased serum levels of brain-derived neurotrophic factor (BDNF) in active treatment groups; a peripheral and non-specific measure. While this is encouraging, further work with more direct and brain-localized measures of neuroplastic effects is urgently needed.

## The future of psychedelic neuroimaging

### Key research questions

The pilot studies mentioned above have put psychedelic research on a firmer and more objective scientific foundation, and have had significant impact in both the scientific community and the popular media [62]. The use of neuroimaging as a tightly-integrated part of the methodology in key clinical trials has facilitated this, not only by providing important scientific results and informing theories of psychedelic effects, but also by producing visually-arresting results (e.g., [21] that have been reproduced extensively in the mainstream media. Portrayals of results like these in the media can often be problematic in terms of misrepresentations and may lead to exaggerated expectations in patients. It is perhaps arguable whether the overall effect of the mainstream media coverage of psychedelic research has been wholly beneficial, however the impact is undeniable.

This general level of raised awareness, coupled with the gradual relaxation of legal restrictions on research in some jurisdictions (driven, at least partly, by the media coverage and the consequent raised awareness), has prompted a psychedelic ‘gold rush’ [63]. Academic research is pressing forward globally at a growing number of research centres, while newly formed companies are seeking to commercialize psychedelic-assisted therapies; with both spheres rapidly expanding the range of compounds used and clinical indications being investigated. In addition, early-stage efforts are underway to develop entirely novel 5-HT2A active compounds [63], including attempts to discover non-hallucinogenic analogues [64, 65]. At the time of writing there are 96 registered clinical trials on the https://www.clinicaltrials.gov/ website containing the search term “psilocybin”, with even larger numbers also registered using “MDMA” (112), and “LSD” (132), as well as some efforts with “Dimethyltryptamine” (20), and “ibogaine” (4). This large number of clinical trials clearly represents intense activity in this space, motivated by the prospect of a new disruptive approach to the treatment of psychiatric disorders. The transdiagnostic nature of psychedelic therapy may provide additional treatment prospects for a wide range of disorders, particularly in such difficult-to-treat conditions as anorexia nervosa [66] that carry high morbidity and mortality. However, the 50-year gap in scientific research arising from legal prohibitions, has led to a severe deficit in the breadth and depth of the basic-science evidence base related to these treatments compared to other commonly-used psychiatric drugs (e.g., SSRI anti-depressants, dopaminergic anti-psychotics).

In our view there are several important outstanding questions which, were they addressed in suitable studies, would represent a significant step forward for the field, and provide a firmer support base for the clinical development of such treatments. These questions are:

1. The relationship between the acute brain effects of psychedelics, and their longer-term (clinically-relevant) effects.
2. The precise effect of psychedelics at the 5-HT2AR, including dose-effect relationships, and how these are related to both acute and longer-term subjective, physiological, and functional effects.
3. The extent to which psychedelics promote neuroplasticity in humans, over what time-scales, and the role neuroplastic processes play in their longer-term (clinically-relevant) effects.

### Key research methods

While in vitro or pre-clinical in vivo work can help address some of these questions, the limitations of animal models, including important differences in the structures of the 5-HT2AR, its binding with psychedelics in rodents versus humans [63], and potential species differences in brain penetrance [67] mean that studies with human subjects are required for a full assessment of these compounds. Fortunately, this modern ‘second-wave’ of psychedelic research can take advantage of modern neuroimaging (and other) technologies to address all these issues in a robust manner in human subjects. MRI is a mature and widely available imaging method which is sensitive to pharmacological effects [68–70]. Recent technical innovations in MRI technology such as accelerated scanning with ‘multiband’ sequences [71], increased signal-to-noise with multi-echo sequences [72], and standardised processing pipelines [73] provide additional capabilities and rigour for this technique. Molecular imaging with the 5-HT2A agonist PET ligand [11C]CIMBI-36 can help elucidate the links between dose and (both acute, and longer-term) clinically-relevant effects [35, 36].

To assess neuroplasticity changes at a cellular/molecular level, the use of recently characterised PET ligands focusing on the synaptic glycoprotein 2A (SV2A, a marker of pre-synaptic terminals; [11C]UCB-J) and mitochondrial complex 1 (MC1, a marker of mitochondrial density; [18F]BCPP-EF) offer promising prospects [74–76]. SV2A is ubiquitously expressed on synaptic terminals, regulates neurotransmitter release, and has a good correlation with established markers of synaptic density such as synaptophysin [74, 77]. While no obvious marker of post-synaptic dendritic spines is currently evaluable using a specific PET radioligand, the preponderance of mitochondria in the post-synaptic over the presynaptic terminals [78] offers the interesting possibility that the use of [11C]UCB-J and [18F]BCPP-EF may provide information about changes in both pre- and post-synaptic terminals. Several MRI-based techniques could also be employed to complement PET methods to assess neuroplasticity changes. These include diffusion tensor imaging (which can provide information about structural connectivity as well as microstructural tissue properties), magnetic resonance spectroscopy (MRS) for quantifying changes in metabolite levels, and various types of functional MRI (principally, task and resting-state paradigms; [79]). MRI-based methods are more indirect measures of neuroplasticity changes than PET, but could provide complementary information on, for instance, functional effects related to underlying molecular/synaptic changes.

### Study proposals

Specifically, regarding question 1, to our knowledge no published study has so far examined both acute and longer-term effects on the brain in the same cohort. Some studies have identified relationships between subjective or questionnaire measures of acute effects and post-treatment neural responses (e.g., [48]). However, directly testing the relationship between the network-disintegration seen in acute fMRI studies [21] with longer-term measures (of neuroplasticity, emotional function, or any of the other previously identified post-dosing changes, both neurological and behavioural/clinically-relevant) would be a crucial test of current theories of psychedelic effects [23, 58].

Question 2 is a related issue, where [11C]CIMBI-36 PET could be used to establish the relationship between drug plasma concentration and 5-HT2AR occupancy (as in [36]). Such information is typically acquired using an adaptive design and through evaluating a time-course of occupancy following a single dose. This allows the estimation of the relationship between plasma concentration and 5-HT2AR occupancy following repeat-dose administration, and is considered to be obligatory data in determining dosing for Phase II and Phase III studies in modern CNS drug development (see [80] for an example). Optimally, such a study could be performed on the new generation of combined PET/MR clinical scanners [81, 82] for simultaneous acquisition of PET and MRI data during acute dosing of a psychedelic compound, with additional follow-up multi-modal scans to assess longer-term effects on neuroplasticity. Multi-modal imaging with PET and (f)MRI would provide information on the molecular-functional-clinical translational bridge, which could potentially help define further novel treatment approaches and new targets for future drug development.

Question 3 would also ideally be addressed with combined PET and MRI methods, but using the metabolic and synaptic-density PET ligands previously mentioned [74], plus complementary MRI-based measures of plasticity, with assessment at multiple time-points. What kind of time-scales these effects should be assessed over is perhaps an open question, but recent work has shown that clinical effects have a rapid onset within 24–48 h and can persist for up to one year post-dosing [83].

For all the studies outlined above, certain ancillary measures and assessments will be critical in developing our understanding of both classic and novel psychedelic drugs. Measurement of drug plasma concentration is critical for the comparison of dosing protocols and routes of administration. In combination with the concentration-occupancy relationship derived from a PET occupancy study, these data will also allow a like-for-like comparison between different psychedelic compounds. Moreover, questionnaires developed and used in previous psychedelic drug trials [84, 85], or even additional brain-imaging technologies such as MEG [18] or EEG-based measures of neuroplasticity (recently shown to be sensitive to drug effects; [86–88]) could also be used, in addition to the primary imaging techniques. Genetic polymorphisms in the 5-HT2AR have been shown to affect some personality and cognitive factors [89, 90], clinical conditions [91] and response to psychiatric medications [92]. Genotyping of polymorphisms in the 5-HT2AR or other relevant molecular targets in the psychedelic response pathway may therefore provide useful information to enable a reduction of variability in other outcome parameters, provide useful hints to underlying biological substrates, or potentially provide means to stratify patients in future studies. The combination of these ancillary measures with multimodal neuroimaging would provide extremely rich data-sets and enable a great number of instructive outcomes and relationships to be assessed. This in turn would provide a platform for future studies which could be focused on assessing the more detailed causal linkages between these factors.

While the proposed studies are primarily focused on the brain, they may also provide wider insights. The highly-potent action of LSD at the BDNF TrkB receptor has recently been highlighted in pre-clinical work [93], with other work showing potent anti-inflammatory effects of psychedelics in animal disease models [94], and effects on gene expression [95]. Taken together, these results suggest psychedelics may have a wide range of effects on systemic biological variables including neurotrophic factors, inflammatory markers, epigenetic features, and possibly others. Investigation of these effects alongside the proposed neuroimaging studies (using appropriate blood/tissue sampling) should also be a high priority. The relationship between the acute/longer-term brain effects and potential changes in these circulating biomarkers is currently unknown, but such investigations may lead to insights into the role these systemic biological changes may have in the therapeutic response.

The studies outlined above are undeniably ambitious. They would require significant resources and investment to accomplish, as well as the use of cutting-edge and somewhat limitedly available technology (e.g., combined PET/MR imaging systems, relatively novel PET ligands). Nevertheless, they are feasible with current methods, and achievable over a reasonable timescale. We have focused on studies with the 5-HT2AR agonist ‘classic’ psychedelics (psilocybin, LSD, DMT), but analogous questions and study outlines could be framed around other novel therapies (ketamine, MDMA, ibogaine etc). with appropriate modifications to the methods (e.g., different PET ligands specific to the pharmacology of the compounds, other MRI modalities, or particular tasks/stimuli). We have also assumed here that these studies would essentially be analogous to early-phase (I/IIa) clinical trials and use healthy subjects, but follow-up studies with clinical populations or inclusion of some of these measures in later-phase clinical trials would also be highly desirable. Care would need to be taken in such cases to minimise the impact of the neuroscientific research on the therapy protocol. These follow-up studies might be particularly valuable in defining biomarkers or neurophenotypes (based on multi-modal imaging) and their relationships to treatment response, in order to stratify patients and deliver personalized treatments. As a possible example, a current issue of debate is the extent to which conventional psychiatric treatments such as SSRIs are likely to interact with psychedelic drugs in patient populations [96]. This is a complicated problem as the interaction can conceivably occur at a number of levels, including peripheral pharmacokinetic interaction (which could be monitored using standard blood pharmacokinetics), central pharmacokinetic interactions (such as competition at the 5-HT2AR between the drug and endogenous serotonin, or antidepressants that have a clinically significant affinity for the 5-HT2AR), or pharmacodynamic interactions such as synergistic or antagonistic effects on second messenger systems or neuroplasticity. Evaluation on each of these levels will require specific protocols that lie beyond the scope of this paper, but the studies proposed here would provide a solid methodological platform and greatly help to inform such efforts. An interesting ‘precision psychiatry’ approach to psychedelic clinical research has recently been proposed by [97]. These authors suggest that the variability in individual response (both the acute, and longer-term clinical responses) could be captured by computational approaches which leverage resting-state fMRI data combined with population-level maps of the 5-HT2AR and gene expression data. These data could then theoretically be used to predict responses to treatments, stratify patients in clinical trials, and select the most appropriate treatments and doses for an individual patient. The authors list a number of pre-requisites for the success of this approach, including: (a) larger study samples, perhaps orders of magnitude larger than extant data, (b) studies which follow-up patients over longer periods, (c) studies which use multiple drugs and multiple patient cohorts with different conditions, and (d) standardised acquisition and analysis methods. These are not likely to be met in the short-term, however the studies proposed herein may be helpful in providing accurate maps of 5-HT2A receptor distribution (or other relevant PET measures), in providing an expanded database of (f)MRI data, and in the use of the various ancillary measures outlined above. The aims of Moujaes et al. are highly commendable, however the history of truly useful (at an individual level) imaging-based biomarkers in psychiatry is, at best, mixed (see [98] for a recent review). The working feasibility of such an approach therefore remains to be seen, but the studies proposed here may be a highly useful initial step along the road to a true precision psychiatry paradigm.

## Novel or ‘second generation’ 5-HT2A compounds

Extensive efforts are currently underway to develop novel 5-HT2AR agonists and test their clinical effects [63]. Recently a prominent psychedelic researcher [99] has warned of the danger of ‘psychedelic exceptionalism’, or the belief that psychedelics are so uniquely powerful and important that they are not bound by the normal rules of clinical governance, ethics, or science. We agree this is a concern, and clearly these novel compounds should be approached using similar paradigms to those developed and used successfully for other novel central nervous system (CNS) drug candidates over the last 50 years. As such, a translational framework for assessing novel neurologically-active compounds that has become established in the field in recent years is the “three pillars” approach. This was first outlined by [100] and subsequently further developed with specific reference to neuroimaging methods [101], the three pillars being: (1) tissue exposure (2) target engagement, and (3) pharmacologic activity. Tissue exposure (does the compound enter the brain in clinically significant concentrations at clinically tolerable doses?) can be established by radiolabelling the drug with a suitable radionuclide and conducting a PET biodistribution study [102–104]. Target engagement (does the compound bind to the target receptor?) can be assessed with [11 C]Cimbi-36 PET to determine 5-HT2AR occupancy and any offsite binding that may affect the tolerability or safety of new compounds can be assessed using other suitable PET radioligands.

The third “pillar” (pharmacologic activity; does the drug cause downstream effects on physiology, brain activity, or some other relevant measure?) can be assessed in a number of ways, most obviously in the current case, putative biomarkers of psychedelic response such as brain network segregation/modularity measured with fMRI [21, 50] and/or PET neuroplasticity changes. In addition to being consistent with general procedures carried out in clinical drug development, this workflow may provide us with key insights into the unique molecular and functional effects of these novel compounds for use in basic-science research and the future development of pharmaceutical drug candidates.

## The role of psychotherapy

The roadmap outlined herein is concerned largely with foundational neuropsychopharmacology, with the most appropriate study participants likely being healthy subjects. However, as noted above, neuroimaging has so far played an important role in some clinical trials of psychedelic therapy (e.g., [50, 53]) and will likely continue to do so. Modern psychedelic therapy is conceptualised by many as “psychedelic-assisted psychotherapy” where the therapeutic benefit is thought to come from a synergistic combination of the drug and accompanying psychotherapy [105, 106]. However, this perspective has recently been challenged by others, asserting the primacy of the drug effects in the therapeutic action [107]. It is relatively well-established that psychotherapy of various types can produce measurable changes in brain function (recent reviews: [108, 109]). A worthwhile aim of clinical (neuroimaging) studies would therefore be to attempt to disentangle the contributions of the drug and therapy components in driving both neurological and clinical effects. Such investigations would need to be relatively large-scale, multi-arm experiments that aim to control multiple aspects of the patient experience in a systematic manner; a serious challenge. Detailed discussion of these issues is well outside the main scope of this proposal, however this is an area that is likely to be hotly debated and intensively investigated as these treatments develop further, and neuroimaging may well play an important role in such studies.

## Conclusions

Neuroimaging technology has played a key part in the modern understanding and development of psychedelic therapies and will likely continue to do so. Our purpose in this perspective piece has been to outline current findings and chart a way forward to address several issues of fundamental importance in the further development of these drugs and associated therapies. Broadly, these studies aim to establish a conceptual ‘molecular-functional-clinical bridge’ by characterising the relationship between acute and longer-term (clinically-relevant) effects, investigation of the 5-HT2A receptor and its association with functional and clinical effects, and investigations focused on neuroplasticity. These studies rely on multimodal neuroimaging (PET and MRI) as a core set of technologies, combined with ancillary measures (e.g. genotyping, subjective measures, pharmacokinetics, EEG/MEG). Neuroimaging methods adapted from the development of other CNS/psychiatric therapies have a lot to offer in this space and should be adopted. While ambitious, these studies are perfectly tractable, and would provide a solid basic-science foundation for the further development of these therapies.

## References

[1] Hardy K (2021). Paleomedicine and the Evolutionary Context of Medicinal Plant Use. Rev Brasileira de Farmacogn.

[2] Sessa B. The History of Psychedelics in Medicine. In: von Heyden M, Jungaberle H, Majić T, editors. Handbuch Psychoaktive Substanzen. Berlin, Heidelberg: Springer; 2016. p. 1–26.

[3] Ban TA (2007). Fifty years chlorpromazine: A historical perspective. Neuropsychiatr Dis Treat.

[4] Ramachandraih CT, Subramanyam N, Bar KJ, Baker G, Yeragani VK (2011). Antidepressants: From MAOIs to SSRIs and more. Indian J Psychiatry.

[5] Nichols DE, Walter H (2021). The History of Psychedelics in Psychiatry. Pharmacopsychiatry.

[6] Dyck E (2005). Flashback: Psychiatric experimentation with LSD in historical perspective. Can J Psychiatry.

[7] Golub A, Bennett AS, Elliott L, Golub A, Bennett AS, Elliott L (2015). Beyond Americas war on drugs: developing public policy to navigate the prevailing pharmacological revolution. AIMS Public Health.

[8] Oram M (2014). Efficacy and Enlightenment: LSD Psychotherapy and the Drug Amendments of 1962. J Hist Med Allied Sci.

[9] Nutt DJ, King LA, Nichols DE (2013). Effects of Schedule I drug laws on neuroscience research and treatment innovation. Nat Rev Neurosci.

[10] Fink M (1969). EEG and Human Psychopharmacology. Annu Rev Pharmacol.

[11] Glennon RA, Titeler M, McKenney JD (1984). Evidence for 5-HT2 involvement in the mechanism of action of hallucinogenic agents. Life Sci.

[12] Vollenweider FX, Vollenweider-Scherpenhuyzen MFI, Bäbler A, Vogel H, Hell D (1998). Psilocybin induces schizophrenia-like psychosis in humans via a serotonin-2 agonist action. NeuroReport.

[13] González-Maeso J, Yuen T, Ebersole BJ, Wurmbach E, Lira A, Zhou M (2003). Transcriptome Fingerprints Distinguish Hallucinogenic and Nonhallucinogenic 5-Hydroxytryptamine 2A Receptor Agonist Effects in Mouse Somatosensory Cortex. J Neurosci.

[14] Rucker JJH, Iliff J, Nutt DJ (2018). Psychiatry & the psychedelic drugs. Past, present & future. Neuropharmacology.

[15] Mithoefer MC, Wagner MT, Mithoefer AT, Jerome L, Doblin R (2010). The safety and efficacy of ±3,4-methylenedioxymethamphetamine- assisted psychotherapy in subjects with chronic, treatment-resistant posttraumatic stress disorder: The first randomized controlled pilot study. J Psychopharmacol.

[16] Carhart-Harris RL, Erritzoe D, Williams T, Stone JM, Reed LJ, Colasanti A (2012). Neural correlates of the psychedelic state as determined by fMRI studies with psilocybin. Proc Natl Acad Sci USA.

[17] Preller KH, Duerler P, Burt JB, Ji JL, Adkinson B, Stämpfli P (2020). Psilocybin Induces Time-Dependent Changes in Global Functional Connectivity. Biol Psychiatry.

[18] Carhart-Harris RL, Muthukumaraswamy S, Roseman L, Kaelen M, Droog W, Murphy K (2016). Neural correlates of the LSD experience revealed by multimodal neuroimaging. Proc Natl Acad Sci.

[19] Preller KH, Burt JB, Ji JL, Schleifer CH, Adkinson BD, Stämpfli P (2018). Changes in global and thalamic brain connectivity in LSD-induced altered states of consciousness are attributable to the 5-HT2A receptor. eLife.

[20] Carhart-Harris RL, Kevin M, Robert L, David E, Wall MB, Bart F (2015). The Effects of Acutely Administered 3,4-Methylenedioxymethamphetamine on Spontaneous Brain Function in Healthy Volunteers Measured with Arterial Spin Labelling and Blood Oxygen Level-Dependent Resting-State Functional Connectivity. Biol Psychiatry.

[21] Petri G, Expert P, Turkheimer F, Carhart-Harris R, Nutt D, Hellyer PJ (2014). Homological scaffolds of brain functional networks. J R Soc Interface.

[22] Luppi AI, Carhart-Harris RL, Roseman L, Pappas I, Menon DK, Stamatakis EA (2021). LSD alters dynamic integration and segregation in the human brain. NeuroImage.

[23] Carhart-Harris RL, Friston KJ (2019). REBUS and the anarchic brain: Toward a unified model of the brain action of psychedelics. Pharmacol Rev.

[24] Geyer MA, Vollenweider FX (2008). Serotonin research: contributions to understanding psychoses. Trends Pharmacol Sci.

[25] Doss MK, Madden MB, Gaddis A, Nebel MB, Griffiths RR, Mathur BN (2022). Models of psychedelic drug action: modulation of cortical-subcortical circuits. Brain.

[26] Vollenweider FX, Geyer MA (2001). A systems model of altered consciousness: integrating natural and drug-induced psychoses. Brain Res Bull.

[27] Kaelen M, Lorenz R, Barrett F, Roseman L, Orban C, Santos-Ribeiro A, et al. Effects of LSD on music-evoked brain activity. bioRxiv. 2017. 10.1101/153031.

[28] Kaelen M, Roseman L, Kahan J, Santos-Ribeiro A, Orban C, Lorenz R, et al. LSD modulates music-induced imagery via changes in parahippocampal connectivity. European Neuropsychopharmacology. 2016. 10.1016/j.euroneuro.2016.03.018. 10.1016/j.euroneuro.2016.03.018 27084302

[29] Preller KH, Pokorny T, Hock A, Kraehenmann R, Stãmpfli P, Seifritz E (2016). Effects of serotonin 2A/1A receptor stimulation on social exclusion processing. Proc Natl Acad Sci USA.

[30] Carhart-Harris RL, Wall MB, Erritzoe D, Kaelen M, Ferguson B, De Meer I (2014). The effect of acutely administered MDMA on subjective and BOLD-fMRI responses to favourite and worst autobiographical memories. Int J Neuropsychopharmacol.

[31] Cumming P, Scheidegger M, Dornbierer D, Palner M, Quednow BB, Martin-Soelch C (2021). Molecular and functional imaging studies of psychedelic drug action in animals and humans. Molecules.

[32] Ettrup A, Holm S, Hansen M, Wasim M, Santini MA, Palner M (2013). Preclinical Safety Assessment of the 5-HT2A Receptor Agonist PET Radioligand [11C]Cimbi-36. Mol Imaging Biol.

[33] Ettrup A, da Cunha-Bang S, McMahon B, Lehel S, Dyssegaard A, Skibsted AW (2014). Serotonin 2A receptor agonist binding in the human brain with [^11^C]Cimbi-36. J Cereb Blood Flow Metab.

[34] Finnema SJ, Stepanov V, Ettrup A, Nakao R, Amini N, Svedberg M (2014). Characterization of [11C]Cimbi-36 as an agonist PET radioligand for the 5-HT2A and 5-HT2C receptors in the nonhuman primate brain. NeuroImage.

[35] Erritzoe D, Ashok AH, Searle GE, Colasanti A, Turton S, Lewis Y (2020). Serotonin release measured in the human brain: a PET study with [11C]CIMBI-36 and d-amphetamine challenge. Neuropsychopharmacology.

[36] Madsen MK, Fisher PM, Burmester D, Dyssegaard A, Stenbaek DS, Kristiansen S, et al. Psychedelic effects of psilocybin correlate with serotonin 2 A receptor occupancy and plasma psilocin levels. Neuropsychopharmacology. 2019. 10.1038/s41386-019-0324-9. 10.1038/s41386-019-0324-9 PMC6785028 30685771

[37] Vollenweider FX, Vontobel P, Hell D, Leenders KL (1999). 5-HT Modulation of Dopamine Release in Basal Ganglia in Psilocybin-Induced Psychosis in Man—A PET Study with [11C]raclopride. Neuropsychopharmacology.

[38] Gouzoulis-Mayfrank E, Schreckenberger M, Sabri O, Arning C, Thelen B, Spitzer M (1999). Neurometabolic effects of psilocybin, 3,4-methylenedioxyethylamphetamine (MDE) and D-methamphetamine in healthy volunteers: A double-blind, placebo-controlled PET study with [18F]FDG. Neuropsychopharmacology.

[39] Madsen MK, Fisher PMD, Stenbæk DS, Kristiansen S, Burmester D, Lehel S (2020). A single psilocybin dose is associated with long-term increased mindfulness, preceded by a proportional change in neocortical 5-HT2A receptor binding. Eur Neuropsychopharmacol.

[40] Stenbæk DS, Madsen MK, Ozenne B, Kristiansen S, Burmester D, Erritzoe D (2021). Brain serotonin 2A receptor binding predicts subjective temporal and mystical effects of psilocybin in healthy humans. J Psychopharmacol.

[41] Erritzoe D, Frokjaer VG, Holst KK, Christoffersen M, Johansen SS, Svarer C (2011). In vivo imaging of cerebral serotonin transporter and serotonin 2A receptor binding in 3,4-methylenedioxymethamphetamine (MDMA or ‘Ecstasy’) and hallucinogen users. Arch Gen Psychiatry.

[42] Carhart-Harris RL, Bolstridge M, Rucker J, Day CMJ, Erritzoe D, Kaelen M (2016). Psilocybin with psychological support for treatment-resistant depression: an open-label feasibility study. Lancet Psychiatry.

[43] Davis AK, Barrett FS, May DG, Cosimano MP, Sepeda ND, Johnson MW (2021). Effects of Psilocybin-Assisted Therapy on Major Depressive Disorder: A Randomized Clinical Trial. JAMA Psychiatry.

[44] Carhart-Harris R, Giribaldi B, Watts R, Baker-Jones M, Murphy-Beiner A, Murphy R (2021). Trial of Psilocybin versus Escitalopram for Depression. N. Engl J Med.

[45] Carhart-Harris RL, Roseman L, Bolstridge M, Demetriou L, Pannekoek JN, Wall MB (2017). Psilocybin for treatment-resistant depression: fMRI-measured brain mechanisms. Sci Rep.

[46] Roseman L, Demetriou L, Wall MB, Nutt DJ, Carhart-Harris RL (2018). Increased amygdala responses to emotional faces after psilocybin for treatment-resistant depression. Neuropharmacology.

[47] Mertens LJ, Wall MB, Roseman L, Demetriou L, Nutt DJ, Carhart-Harris RL. Therapeutic mechanisms of psilocybin: Changes in amygdala and prefrontal functional connectivity during emotional processing after psilocybin for treatment-resistant depression. J Psychopharmacol. 2020;34:1–14. 10.1177/0269881119895520 31941394

[48] Wall MB, Lam C, Ertl N, Kaelen M, Roseman L, Nutt DJ (2023). Increased low-frequency brain responses to music after psilocybin therapy for depression. J Affect Disord.

[49] Barrett FS, Doss MK, Sepeda ND, Pekar JJ, Griffiths RR (2020). Emotions and brain function are altered up to one month after a single high dose of psilocybin. Sci Rep.

[50] Daws RE, Timmermann C, Giribaldi B, Sexton JD, Wall MB, Erritzoe D, et al. Increased global integration in the brain after psilocybin therapy for depression. Nature Medicine. 2022. 10.1038/s41591-022-01744-z. 10.1038/s41591-022-01744-z 35411074

[51] Doss MK, Barrett FS, Corlett PR (2022). Skepticism about Recent Evidence That Psilocybin ‘Liberates’ Depressed Minds. ACS Chem Neurosci.

[52] Carhart-Harris RL (2019). How do psychedelics work?. Curr Opin Psychiatry.

[53] Doss MK, Považan M, Rosenberg MD, Sepeda ND, Davis AK, Finan PH (2021). Psilocybin therapy increases cognitive and neural flexibility in patients with major depressive disorder. Transl Psychiatry.

[54] McCulloch DE-W, Knudsen GM, Barrett FS, Doss MK, Carhart-Harris RL, Rosas FE (2022). Psychedelic resting-state neuroimaging: A review and perspective on balancing replication and novel analyses. Neurosci Biobehav Rev.

[55] Raval NR, Johansen A, Donovan LL, Ros NF, Ozenne B, Hansen HD (2020). P.804A single dose of psilocybin increases synaptic density and decreases 5-HT2A receptor density in the pig brain. Eur Neuropsychopharmacol.

[56] Aleksandrova LR, Phillips AG (2021). Neuroplasticity as a convergent mechanism of ketamine and classical psychedelics. Trends Pharmacol Sci.

[57] Artin H, Zisook S, Ramanathan D (2021). How do serotonergic psychedelics treat depression: The potential role of neuroplasticity. World J Psychiatry.

[58] Carhart-Harris RL, Nutt DJ (2017). Serotonin and brain function: A tale of two receptors. J Psychopharmacol.

[59] de Vos CMH, Mason NL, Kuypers KPC (2021). Psychedelics and Neuroplasticity: A Systematic Review Unraveling the Biological Underpinnings of Psychedelics. Front Psychiatry.

[60] Hutten NRPW, Mason NL, Dolder PC, Theunissen EL, Holze F, Liechti ME (2021). Low Doses of LSD Acutely Increase BDNF Blood Plasma Levels in Healthy Volunteers. ACS Pharmacol Transl Sci.

[61] de Almeida RN, Galvão ACdeM, da Silva FS, Silva EAdosS, Palhano-Fontes F, Maia-de-Oliveira JP (2019). Modulation of serum brain-derived neurotrophic factor by a single dose of ayahuasca: Observation from a randomized controlled trial. Front Psychol.

[62] Byock I (2018). Taking Psychedelics Seriously. J Palliat Med.

[63] McClure-Begley TD, Roth BL The promises and perils of psychedelic pharmacology for psychiatry. Nature Reviews Drug Discovery. 2022. 10.1038/s41573-022-00421-7. 10.1038/s41573-022-00421-7 35301459

[64] Crunkhorn S (2022). Designing nonhallucinogenic psychedelic analogues. Nat Rev Drug Discov.

[65] Cao D, Yu J, Wang H, Luo Z, Liu X, He L (2022). Structure-based discovery of nonhallucinogenic psychedelic analogs. Science.

[66] Spriggs MJ, Douglass HM, Park RJ, Read T, Danby JL, de Magalhães FJC (2021). Study Protocol for “Psilocybin as a Treatment for Anorexia Nervosa: A Pilot Study”. Front Psychiatry.

[67] Deo AK, Theil FP, Nicolas JM (2013). Confounding parameters in preclinical assessment of blood-brain barrier permeation: An overview with emphasis on species differences and effect of disease states. Mol Pharmaceutics.

[68] Jenkins BG (2012). Pharmacologic magnetic resonance imaging (phMRI): imaging drug action in the brain. NeuroImage.

[69] Bourke JH, Wall MB (2015). phMRI: methodological considerations for mitigating potential confounding factors. Front Neurosci.

[70] Borsook D, Becerra L, Hargreaves R (2006). A role for fMRI in optimizing CNS drug development. Nat Rev Drug Discov.

[71] Demetriou L, Kowalczyk OS, Tyson G, Bello T, Newbould RD, Wall MB (2018). A comprehensive evaluation of increasing temporal resolution with multiband-accelerated sequences and their effects on statistical outcome measures in fMRI. NeuroImage.

[72] Todd N, Josephs O, Zeidman P, Flandin G, Moeller S (2017). Functional Sensitivity of 2D Simultaneous Multi-Slice Echo-Planar Imaging: Effects of Acceleration on g-factor and Physiological Noise. Front Neurosci.

[73] Esteban O, Markiewicz C, Blair RW, Moodie C, Isik AI, Aliaga AE, et al. FMRIPrep: a robust preprocessing pipeline for functional MRI. bioRxiv. 2018:306951. 10.1038/s41592-018-0235-4 PMC6319393 30532080

[74] Mansur A, Rabiner EA, Comley RA, Lewis Y, Middleton LT, Huiban M (2020). Characterization of 3 PET tracers for quantification of mitochondrial and synaptic function in healthy human brain: 18F-BCPP-EF, 11C-SA-4503, and 11C-UCB-J. J Nucl Med.

[75] Tsukada H, Nishiyama S, Fukumoto D, Kanazawa M, Harada N (2014). Novel PET Probes 18F-BCPP-EF and 18F-BCPP-BF for Mitochondrial Complex I: A PET Study in Comparison with 18F-BMS-747158-02 in Rat Brain. J Nucl Med.

[76] Finnema SJ, Nabulsi NB, Eid T, Detyniecki K, Lin SF, Chen MK (2016). Imaging synaptic density in the living human brain. Sci Transl Med.

[77] Nowack A, Yao J, Custer KL, Bajjalieh SM (2010). SV2 regulates neurotransmitter release via multiple mechanisms. Am J Physiol - Cell Physiol.

[78] Santuy A, Turégano-López M, Rodríguez JR, Alonso-Nanclares L, Defelipe J, Merchán-Pérez A (2018). A Quantitative Study on the Distribution of Mitochondria in the Neuropil of the Juvenile Rat Somatosensory Cortex. Cereb Cortex.

[79] Hamaide J, De Groof G, Van der Linden A (2016). Neuroplasticity and MRI: A perfect match. NeuroImage.

[80] Rabiner EA, Beaver J, Makwana A, Searle G, Long C, Nathan PJ (2011). Pharmacological differentiation of opioid receptor antagonists by molecular and functional imaging of target occupancy and food reward-related brain activation in humans. Mol Psychiatry.

[81] Wehrl HF, Hossain M, Lankes K, Liu C-C, Bezrukov I, Martirosian P (2013). Simultaneous PET-MRI reveals brain function in activated and resting state on metabolic, hemodynamic and multiple temporal scales. Nat Med.

[82] Mansur A, Newbould R, Searle GE, Redstone C, Gunn RN, Hallett WA (2018). PET-MR Attenuation Correction in Dynamic Brain PET Using [11C]Cimbi-36: A Direct Comparison with PET-CT. IEEE Trans Radiat Plasma Med Sci.

[83] Gukasyan N, Davis AK, Barrett FS, Cosimano MP, Sepeda ND, Johnson MW (2022). Efficacy and safety of psilocybin-assisted treatment for major depressive disorder: Prospective 12-month follow-up. J Psychopharmacol (Oxf, Engl).

[84] Nour MM, Evans L, Nutt D, Carhart-Harris RL (2016). Ego-dissolution and psychedelics: Validation of the ego-dissolution inventory (EDI). Front Hum Neurosci.

[85] Roseman L, Haijen E, Idialu-Ikato K, Kaelen M, Watts R, Carhart-Harris R (2019). Emotional breakthrough and psychedelics: Validation of the Emotional Breakthrough Inventory. J Psychopharmacol.

[86] Loheswaran G, Barr MS, Zomorrodi R, Rajji TK, Blumberger DM, Foll BL (2017). Impairment of Neuroplasticity in the Dorsolateral Prefrontal Cortex by Alcohol. Sci Rep.

[87] Salavati B, Daskalakis ZJ, Zomorrodi R, Blumberger DM, Chen R, Pollock BG (2018). Pharmacological modulation of long-term potentiation-like activity in the dorsolateral prefrontal cortex. Front Hum Neurosci.

[88] Sumner RL, McMillan R, Spriggs MJ, Campbell D, Malpas G, Maxwell E (2020). Ketamine Enhances Visual Sensory Evoked Potential Long-term Potentiation in Patients With Major Depressive Disorder. Biol Psychiatry: Cogn Neurosci Neuroimaging.

[89] Fiocco AJ, Joober R, Poirier J, Lupien S (2007). Polymorphism of the 5-HT2A receptor gene: Association with stress-related indices in healthy middle-aged adults. Front Behav Neurosci.

[90] Gong P, Liu J, Blue PR, Li S, Zhou X (2015). Serotonin receptor gene (HTR2A) T102C polymorphism modulates individuals’ perspective taking ability and autistic-like traits. Front Hum Neurosci.

[91] Qin B, Sun Z, Liang Y, Yang Z, Zhong R (2014). The Association of 5-HT2A, 5-HTT, and LEPR Polymorphisms with Obstructive Sleep Apnea Syndrome: A Systematic Review and Meta-Analysis. PLOS ONE.

[92] Olajossy-Hilkesberger L, Godlewska B, Schosser-Haupt A, Olajossy M, Wojcierowski J, Landowski J (2011). Polymorphisms of the 5-HT2A receptor gene and clinical response to olanzapine in paranoid schizophrenia. Neuropsychobiology.

[93] Moliner R, Girych M, Brunello CA, Kovaleva V, Biojone C, Enkavi G (2023). Psychedelics promote plasticity by directly binding to BDNF receptor TrkB. Nat Neurosci.

[94] Flanagan TW, Nichols CD (2018). Psychedelics as anti-inflammatory agents. Int Rev Psychiatry.

[95] Martin DA, Nichols CD. The Effects of Hallucinogens on Gene Expression. In: Halberstadt AL, Vollenweider FX, Nichols DE, editors. Behavioral Neurobiology of Psychedelic Drugs, Berlin, Heidelberg: Springer; 2018. p. 137–58.

[96] Sarparast A, Thomas K, Malcolm B, Stauffer CS. Drug-drug interactions between psychiatric medications and MDMA or psilocybin: a systematic review. Psychopharmacology. 2022;239:1945–76 10.1007/s00213-022-06083-y PMC9177763 35253070

[97] Moujaes F, Preller KH, Ji JL, Murray JD, Berkovitch L, Vollenweider FX, et al. Towards mapping neuro-behavioral heterogeneity of psychedelic neurobiology in humans. Biol. Psychiatry. 2022. 10.1016/j.biopsych.2022.10.021. 10.1016/j.biopsych.2022.10.021 36715317

[98] Abi-Dargham A, Horga G (2016). The search for imaging biomarkers in psychiatric disorders. Nat Med.

[99] Johnson MW (2021). Consciousness, Religion, and Gurus: Pitfalls of Psychedelic Medicine. ACS Pharmacol Transl Sci.

[100] Morgan P, Van Der Graaf PH, Arrowsmith J, Feltner DE, Drummond KS, Wegner CD (2012). Can the flow of medicines be improved? Fundamental pharmacokinetic and pharmacological principles toward improving Phase II survival. Drug Discov Today.

[101] Gunn RN, Rabiner EA (2017). Imaging in Central Nervous System Drug Discovery. Semin Nucl Med.

[102] Wong D, Tauscher J, Gründer G (2008). The role of imaging in proof of concept for CNS drug discovery and development. Neuropsychopharmacology.

[103] Jones T, Rabiner EA (2012). The development, past achievements, and future directions of brain PET. J Cereb Blood Flow Metab: Off J Int Soc Cereb Blood Flow Metab.

[104] Ridler K, Cunningham V, Huiban M, Martarello L, Pampols-Maso S, Passchier J (2014). An evaluation of the brain distribution of [11C]GSK1034702, a muscarinic-1 (M1) positive allosteric modulator in the living human brain using positron emission tomography. EJNMMI Res.

[105] Greenway KT, Garel N, Jerome L, Feduccia AA (2020). Integrating psychotherapy and psychopharmacology: psychedelic-assisted psychotherapy and other combined treatments. Expert Rev Clin Pharmacol.

[106] Watts R, Luoma JB (2020). The use of the psychological flexibility model to support psychedelic assisted therapy. J Contextual Behav Sci.

[107] Goodwin GM, Malievskaia E, Fonzo GA, Nemeroff CB Must Psilocybin Always “Assist Psychotherapy”? AJP. 2023:appi.ajp.20221043. 10.1176/appi.ajp.20221043 37434509

[108] Barsaglini A, Sartori G, Benetti S, Pettersson-Yeo W, Mechelli A (2014). The effects of psychotherapy on brain function: A systematic and critical review. Prog Neurobiol.

[109] Beauregard M (2014). Functional neuroimaging studies of the effects of psychotherapy. Dialogues Clin Neurosci.

